# Role of Statin Therapy in Primary Prevention of Cardiovascular Disease in Elderly Patients

**DOI:** 10.1007/s11883-019-0793-7

**Published:** 2019-05-20

**Authors:** Timo E. Strandberg

**Affiliations:** 1 University of Helsinki, PO Box 340, FIN-00029 Helsinki, Finland; 2 Center for Life Course Health Research, University of Oulu, Oulu, Finland; 3 Helsinki University Hospital, Helsinki, Finland

**Keywords:** Aged, Cardiovascular, Statin, Primary prevention

## Abstract

**Purpose of Review:**

Hypercholesterolemia and statin treatment are nowadays common among people older than 75 years, but clinical heterogeneity in this increasing age group is wide, and treatment decisions may differ from those in younger patients. Aim is to discuss the presentation, modifying factors, and treatment decisions of hypercholesterolemia (usually with statins) in older persons and focusing on primary prevention.

**Recent Findings:**

There are no randomized controlled trials in persons older than 80 years at baseline. Randomized controlled trial findings in younger patients and 75+ subgroups and in observational studies support treatment in secondary prevention of atherosclerotic cardiovascular disease (ASCVD), but trial evidence in primary prevention is less clear. Available data do not imply specific harms in older patients, and, therefore, also, judicious primary prevention is possible. However, persons older than 75 years are biologically a very heterogeneous group with frequent frailty, comorbid conditions, and multiple concomitant drugs. All these, as well as personal preferences, must be taken into account in treatment decisions.

**Summary:**

Statin treatment is only one way to prevent ASCVD in older people. Treatment of hypercholesterolemia should be started far before 75–80 years, and there is no need to discontinue statin treatment due to chronological age alone. After 75 years, treatment should be started in patients with ASCVD and judiciously in primary prevention. Like all prevention, statin treatment should be discontinued when palliative treatment is started. Ongoing and planned trials in 70+ individuals will give more information about primary prevention in older persons.

## Introduction

Patients aged 75–80 years and over are an increasing group of people in aging societies worldwide and, also, an increasing target of prevention. Because prevention requires a certain amount of life expectancy to be relevant, it is important to realize that the mean life expectancy for an 80-year-old woman is between 7.4 and 10.6 years and for an 80-year-old man between 6.3 and 8.6 years (http://stats.oecd.org/Index.aspx/DataSetCode=HEALTH_STAT). It is also important to consider the overall functioning and quality of life of older people, and there are several lines of evidence that these have been improving. For example, a study comparing groups of people born in 1905 or 1915 (ages 93 and 95 years, respectively) indicated that people continue to very old age with better functioning [1]. Consequently, not only curative or palliative but also preventive efforts are getting more and more important among oldest patients.

While smoking cessation plays no more a great role among people aged 75 years and older, important preventive efforts of cardiovascular disease (CVD) include treatment of hypertension and anticoagulation to prevent stroke in atrial fibrillation. Also, hypercholesterolemia is common, and accordingly, use of the inhibitors of 3-hydroxy-3-methylglutaryl coenzyme A (HMG-CoA) reductase (statins) has greatly increased especially during the last decade among the oldest age groups. In a large US survey, the prevalent use was 29, 24, and 14% in the age ranges of 80–84, 85–89, and over 90 years, respectively [2]. On the other hand, older patients often have low adherence and are discontinuing statin treatment, especially in primary prevention [3, 4].

Paradoxically, several epidemiological studies have suggested that in old age low cholesterol is associated with worse prognosis [5], and this has led to concerns and criticism about the relevance of giving statins to older people despite their high cholesterol in primary prevention. The question is important, because the decline in atherosclerotic CVD (ASCVD) mortality before 65 years has led to increased numbers of older patients at risk or with manifest ASCVD, for which LDL cholesterol is the leading causative factor [6••]. Moreover, common geriatric conditions, including dementia and frailty, may have atherosclerotic origins [7, 8••], and arterial aging [9•] further disposes older people to vascular disease.

This narrative review is partly based on an earlier review [10] of the best available evidence up to 2014 of hypercholesterolemia and its treatment in people aged 80 years and over. Focus here is in studies after 2014 and in primary prevention with statins. For secondary prevention, I can concur with the American guideline on the treatment of blood cholesterol [11••], which also includes recommendations for patients older than 75 years.

## General Outline of Hypercholesterolemia Treatment

There is an established and graded association between serum cholesterol and ASCVD risk [6], and usually hypercholesterolemia is considered to be present when total cholesterol exceeds 5 mmol/L on the average corresponding to 3 mmol/L for LDL cholesterol. LDL is the primary, “predisposing” cause of ASCVD, and its lowering with various treatments, like statins and new proprotein convertase subtilisin/kexin type 9 (PCSK9) inhibitors, have been robustly shown to reduce cardiovascular morbidity and mortality (statins), in trials with median age around 60 years [12••, 13•].

Atherosclerotic process in the arterial wall starts to develop at LDL cholesterol levels over 1.8 mmol/L. However, the development of clinical ASCVD events, myocardial infarctions and strokes, in midlife and old age is also influenced by “precipitating” factors (smoking, hypertension, diabetes) superimposed on LDL cholesterol. Therefore, total ASCVD risk is used to indicate the need and mode for hypercholesterolemia treatment [11••]. Although treatment is often started late, it is evident that the oldest statin users have a long-term “cholesterol burden” in their arteries. This should be remembered when older statin users and nonusers are compared.

## Paradox of Low Cholesterol in Old Age

One of the reasons for low adherence of statin treatment in older people may be the aforementioned paradox in epidemiological studies suggesting low cholesterol in old age is associated with increased mortality risk [5]. However, Mendelian randomization studies indicate that high LDL cholesterol level preserves its risk function even in the oldest-old [14].

**Fig. 1:**
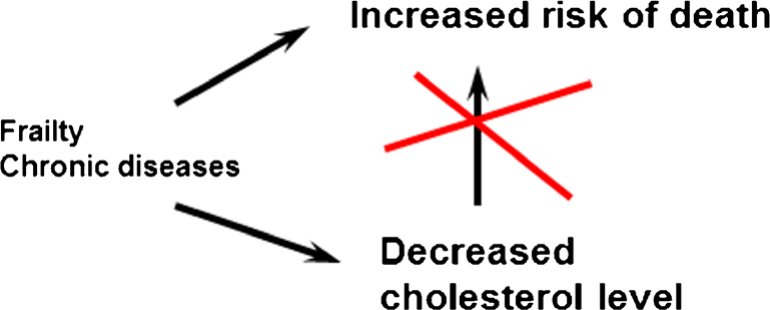
Explanation to the “cholesterol paradox,” that is, worse prognosis with lower cholesterol level in observational studies

For the explanation of the paradox, important confounders affecting both cholesterol level and mortality must be taken into account. In old age and frailty, low serum cholesterol may be a marker of changes in cholesterol metabolism [15], a marker of terminal decline [16], or a marker of subclinical disease, such as cancer [17, 18]. Inflammation is mediated by interleukins (IL-6) which increase LDL receptors leading to lower serum LDL cholesterol levels [19•]. These background factors are the true reason for worse prognosis (Fig. 1). Consequently, “exogenous” (therapeutic) cholesterol-lowering must be differentiated from cholesterol lowering due to internal, “endogenous” mechanisms. Neither is there evidence that current treatments would reduce LDL cholesterol too much, because very low levels (0.5–1.0 mmol/L, even lower) are sufficient for cellular functions [6, 13•].

## Statin Treatment in Older People

When assessing the effects and studies of statins in people older than 75 years, several aspects should be taken into account (Table 1).

**Table 1:** Points to consider about statin treatment in older people

- Is statin treatment ongoing or started after 75 years of age? - What are the individual characteristics: robust vs frail and multimorbid; living in the community vs nursing home residents (who include an increasing proportion of individuals with advanced dementia); short vs long life expectancy. - Role of adverse effects potentially specific for older people. - What is the difference between primary vs secondary prevention in patients over 75 years of age? - What about treatment after 80–85 years?	

### Starting Before or After 75 Years

There is robust evidence that statin treatment—when started in midlife—is beneficial for reducing even total mortality in primary prevention [12••]. It is difficult to comprehend that this effect would vanish after some arbitrary age threshold, and there are several studies where ongoing statin treatment is associated with better prognosis in older people, too [10, 20, 21]. The ACC/AHA guideline supports continuing statin beyond 75 years in persons already taking and tolerating the drug [11••], and an algorithm has been published to guide treatment [10].

It can be claimed that only “deprescribing” studies could confirm the question about benefits of continuing earlier started treatment in old age. Some studies have investigated the effects of discontinuation at the very end of life and showing benefit for quality of life [22]. However, an unblinded study is open to bias. Given the better prognosis with statin treatment in epidemiological studies, also ethical questions should be considered in discontinuation of statin treatment in old age [23]. There is an ongoing trial in France, Statins In The Elderly (SITE, NCT02547883), which is recruiting 2430 participants aged 75 years and over and treated in primary prevention. Among them, statin treatment will be randomly discontinued or continued. The participants will be followed-up every 3 months for a total of 36 months, and clinical events and economic impact were also registered. The trial has started in 2016 and expected to close in 2021.

### Heterogeneity of Older People

People older than 75 years are biologically very heterogeneous, ranging from institutionalized patients with dementia to robust and strong old individuals. They may have features (frailty, multimorbidity, polypharmacy, increased vulnerability to adverse effects, competing mortality, etc.), which usually have not been addressed or such patients excluded in randomized trials, and this must be taken into account when individual prevention is considered. Prevention may also be futile, if started too late—for example, in patients with dementia, late-stage heart, or kidney failure [24–26]. On the other hand, when used in persons with low risk, adverse effects of drugs may exceed benefits.

Age itself is a powerful risk factor due to the accumulation of cellular damage from aging and risk factor burden. Moreover, the predictive value of traditional risk factors—e.g., cholesterol, blood pressure, and obesity—may be reversed in old age (see previous texts). Therefore, focusing primary prevention to those older individuals with most benefit is challenging. Assessment of frailty [27], biomarkers—such as homocysteine [28]—or more conventional indicators of ASCVD, for example, coronary calcium score, carotid intima media thickness, and genetic markers, may offer methods to better assess risk, but their use is not established.

On the other hand, it is reassuring that in epidemiologic studies ongoing statin treatment has been beneficial irrespective of multiprognostic factors [20, 21] and statin efficacy noted in RCTs is similar in “real-life” and with other drug treatments [29]. The significance of potential adverse effects of statins is accentuated in primary prevention, because benefits are smaller (or are achieved later) in people at lower ASCVD risk. These will be discussed next.

### Role of Adverse Effects

Common concerns of statin treatment in older people have included fears of myalgia and myositis, diabetes, cognitive disorders, hemorrhagic stroke, fatigue, and loss of energy leading to less physical activity, worsened quality of life, and drug interactions in patients with polypharmacy. As most of these have not been observed in RCTs, this has been explained by the focus of clinical trials on efficacy endpoints, and, therefore, less serious adverse effects may not have been fully recorded. They would nevertheless be important in frail older people if they lead, for example, to physical inactivity and functional decline. Frail individuals with comorbidity and polypharmacy may also be more prone to adverse effects and drug interactions, and participants with these problems have been usually excluded from clinical trials. Furthermore, serious adverse events may also be more frequent in “real life” conditions than in controlled studies.

The RCT-established and well-known adverse effects of statins have included effects on liver enzymes, musculoskeletal effects, and development of diabetes [12••]. Although several other adverse effects have been suspected, there is seldom consistent evidence of a cause–effect relationship [30, 31].

The muscle-related effects are potentially the most important ones for an older patient. These effects range from pains without serum creatinine kinase (CK) elevations to rhabdomyolysis, but the occurrence of life-threatening toxicity is rare. Milder symptoms, myalgia and cramps, without CK elevations are common in “real-life” settings, but true cause–effect relationship is often difficult to establish. In a large observational study (PRIMO), muscle symptoms were reported by 10.5% [32], which nevertheless would mean a large number of individuals, because statin use is so common.

Among vulnerable older people, adverse muscle effects could promote sarcopenia and predispose to frailty, falls, and morbidity especially in nursing home residents. However, hard evidence for this is absent in available studies [10], and statin treatment has no general deteriorating effect on frailty nor on physical function. At the individual level, it is still important to observe potential adverse effects like drug interactions, dehydration, and comorbidity in a frail patient.

Elevations of hepatic transaminases usually resolve spontaneously, after dose reduction or discontinuation of the drug. Progression to serious liver damage is extremely rare, and routine liver enzyme testing is not needed.

Statin treatment is associated with a slightly increased risk of diabetes, but, in older people, the clinical significance is unclear, and statin treatment also benefits patients with diabetes [12••]. At present, there is no firm evidence that statin treatment would have either positive or negative effect on cognition [12••, 30, 31], and very low cholesterol is not associated with cognitive impairment [33]. Given the established effect of preventing ischemic stroke [12••], a risk factor for cognitive decline, statin treatment could be rather anticipated to be beneficial through that mechanism. Slightly increased risk of hemorrhagic stroke has been noted in patients with prior stroke [12••, 31], but lower risk was recently observed in a survey of a large population without previous stroke [34].

Finally, although RCTs of statins have been negative in advanced heart failure, late-stage renal disease, or dementia, neither have specific harms been detected in these most vulnerable patient groups. In a large real-life database, statins appeared to be similarly tolerated in older (> 75 years) and younger adults in primary prevention [35]. In accordance, health-related quality of life was similar among home-living octogenarian statin users as compared to nonusers [36].

### Statin Treatment Started in Old Age in Primary Prevention

Meta-analysis of statin trials has revealed that each 1 mmol/L reduction in LDL cholesterol decreases the yearly rate of major ASCVD events by one fifth and total mortality by 10% [12••]. The relative reduction of ASCVD has been independent of baseline LDL cholesterol level and of various subgroups. With relative risk of ASCVD being constant and absolute risk higher in older people, statin treatment should lead to benefits also in people aged 75 and older. This is clearly the case for secondary prevention according to a meta-analysis of randomized statin trials [37••]. The meta-analysis included 14,483 participants older than 75 years, and statin treatment was associated with a 15% reduced rate of major vascular events (HR 0.85, 95% CI 0.73–0.98) among those with existing ASCVD.

In primary prevention, direct evidence of benefit in age groups 75 years and over has been less clear in RCTs [37••]. Observational studies up to 2014 have suggested mixed benefit, and recent observational statin studies (mostly > 75 years, some starting from 70 years) and new analyses of older RCTs in primary prevention are shown in Table 2 [38–43]. Overall, the results are not unequivocal but seem to suggest benefits of starting statin in patients older than 75 years at increased risk of ASCVD, such as diabetics.

**Table 2:** Recent observational studies and randomized controlled trials of statins in older people without atherosclerotic cardiovascular disease (ASCVD)

Source	No. of participants with details of age (year)	Mean/median follow-up (year)	Patients	Findings in statin users vs nonusers (or less intensive treatment)
Observational studies				
Orkaby et al. [38]	1130 statin users were matched to nonusers > 70 years Total *n* = 7213	7	Primary prevention	All-cause mortality: HR 0.82, 95% CI 0.69–0.98 CVD events: HR 0.86, 95% CI 0.70–1.06 Stroke: HR 0.70, 95% CI 0.45–1.09 In subgroup analyses, results were similar in age groups at baseline (70–76 or > 76 years) or according to functional status.
Ramos et al. [39]	7502 new statin users were matched to nonusers, > 75 years Total *n* = 46,864	5.6	Primary prevention	*75–84 year olds without diabetes:* ASCVD: HR 0.94, 95% CI 0.86–1.04 All-cause mortality: HR 0.98, 95% CI 0.91–1.05 *85 and older without diabetes* ASCVD: HR 0.93, 0.82–1.06 All-cause mortality: HR 0.97, 95% CI 0.90–1.05 *75–84 year olds with diabetes* ASCVD: HR 0.76, 95% CI 0.65–0.89 All-cause mortality: HR 0.84, 95% CI 0.75–0.94 *85 and older with diabetes* ASCVD: HR 0.82, 95% CI 0.53–1.26 All-cause mortality: HR 1.05, 95% CI 0.86–1.28
Bezin et al. [40]	New statin users matched to nonusers, > 75 years. Total *n* = 7284	4.7	Primary prevention	HR 0.93 (95% CI 0.89–0.96) in people with modifiable risk factors (diabetes or cardiovascular medications). HR 1.01 (95% CI 0.86–1.18) in people without modifiable risk factors
Jun et al. [41]	11,017 > 75 years	Nested case–control	Primary prevention	Composite outcome: adjusted OR [AOR] 0.77; 95% CI 0.71–0.84 Stroke: AOR 0.74; 95% CI 0.61–0.89 All-cause death: AOR 0.73; 95% CI 0.66–0.81
Kim et al. (SCOPE-75) [42]	639 statin users, 639 statin never users, > 75 years	5.2	Primary prevention (but with ASCVD risk factors)	Major adverse cardiovascular and cerebrovascular events: HR 0.59, 95% CI 0.41–0.85. All-cause death: HR 0.56, 95% CI 0.34–0.93.
Randomized controlled trials				
JUPITER	5695 > 70 years	2.2	Primary prevention	Combined cardiovascular end point: HR 0.61; 95% CI 0.43–0.86; *P* = 0.004 All-cause mortality: HR 0.80; 95% CI 0.62–1.0; *P* = 0.09
HOPE-3	3086 > 70 years	5.0	Primary prevention	Combined cardiovascular end point: HR 0.83; 95% CI 0.64–1.07; *P* = 0.16 All-cause mortality: HR 0.91; 95% CI 0.73–1.13; *P* = 0.38
Meta-analysis of JUPITER and HOPE-3 [43]	8781 > 70 years			Composite end point of nonfatal myocardial infarction, nonfatal stroke, or cardiovascular death: HR 0.74, 95% CI 0.61–0.91
Meta-analysis of statin trials in older people [37••]	14,483 > 75 years	4.9	Primary and secondary prevention	Major vascular events: Primary prevention HR 0.92, 95% CI 0.73–1.16 Secondary prevention: HR 0.85, 95% CI 0.73–0.98

A clinical trial, STAtin Therapy for Reducing Events in the Elderly (STAREE, NCT02099123), is currently ongoing and enrolling 18,000 participants for a randomized and placebo-controlled trial to determine whether atorvastatin (40 mg daily) will extend the length of a disability-free life (survival outside permanent residential care) among healthy participants aged 70 years and older. The trial has started in 2015 and is expected to close in 2023. Primary outcome measures of STAREE are either (1) death or development of dementia (measured by cognitive function tests) or development of disability (measured by the KATZ ADL test) or (2) a major fatal or non-fatal CVD event. Numerous secondary outcomes, such as quality of life, cognitive decline, frailty, and cost-effectiveness, will also be analyzed.

### Oldest-Old

There are no randomized studies about starting statin specifically after 80 or 85 years, and the oldest age groups (including only modest number of individuals) are not separated in trials. According to expert groups of the American National Institutes of Aging (NIA) [44], prospective, traditional, placebo-controlled, randomized clinical trials, and pragmatic trials would be suitable options to address gaps in knowledge of oldest patients. Accordingly, the experts urge to include better representation of very old adults, women, underrepresented minorities, and individuals with various health status, cognitive, socioeconomic, and educational backgrounds in future trials. However, it may be challenging to recruit oldest patients for a placebo-controlled trial and it is feared that a potentially negative result (like lack of effect of statins in end-stage disease) would be wrongly applied to those older patients, whose treatment has been started earlier in life, simply because of age [45].

## Conclusions

We have earlier constructed a treatment algorithm for statin treatment in people older than 80 years taking into account whether the treatment is ongoing or considered de novo [10]. Evidence accumulated after 2014 does not give reason to change the general principles in older patients. Statin treatment is generally safe and well-tolerated also in older patients, and ongoing treatment should be continued after 75 years. Starting treatment in primary prevention in older age is indicated, if the patient is considered to be at increased risk for ASCVD. However, individual assessment and shared decision-making (provided the patient has truthful information about statins!) are important, and it is also important to secure adherence. Ongoing trials will give further information in the older patient groups and will be available in the early 2020s.
